# Supplementary figures and images for: Prediction of mortality and prioritisation to tertiary care using the ‘OUR-ARCad’ risk score gleaned from the second wave of COVID-19 pandemic—A retrospective cohort study from South India
Source: PLoS One. 2025 Jan 24;20(1):e0312993. doi: 10.1371/journal.pone.0312993 (PMC11761102; doi:10.1371/journal.pone.0312993)

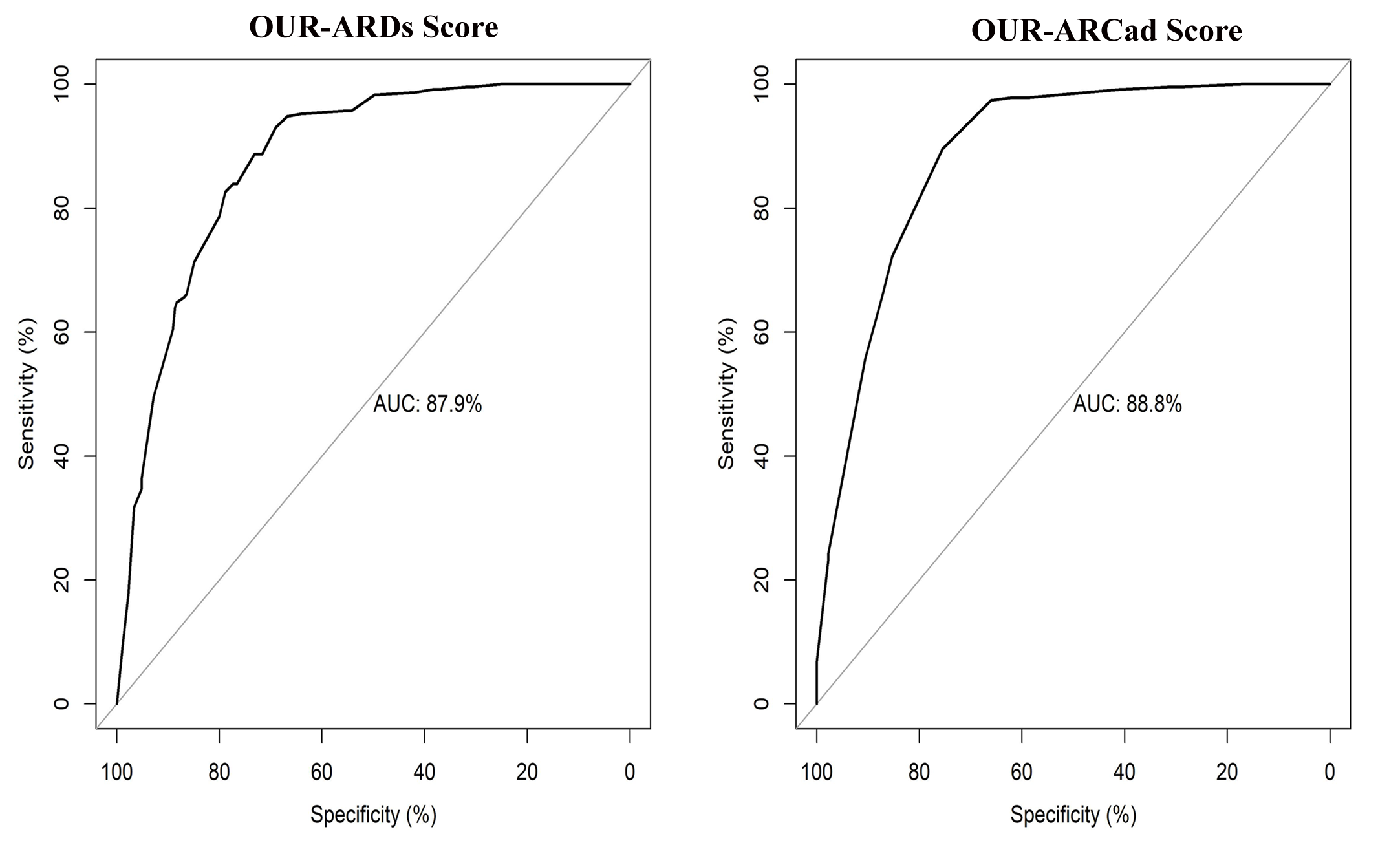

Supplement: S1 Fig — OUR-ARDs score can be found in reference 1 “Gopalan N, Senthil S, Prabakar NL et al. Predictors of mortality among hospitalized COVID-19 patients and risk score formulation for prioritizing tertiary care—An experience from South India. PLoS One [Internet]. 2022;17(2): e0263471. Available from: http://dx.doi.org/10.1371/journal.pone.0263471”. OUR-ARDs and OUR-ARCad: O–peripheral oxygen saturation in percentage, U–urea in milligram per decilitre, R–Neutrophil lymphocyte ratio, A–age in years, R–Pulse rate in beats per minute, D–Diabetes mellitus, Cad–coronary artery disease / cardiovascular disease, %—percentage, AUC–Area under the Curve. (TIF) [file pone.0312993.s001.tif]
