# Supplementary material for: Prediction of mortality and prioritisation to tertiary care using the ‘OUR-ARCad’ risk score gleaned from the second wave of COVID-19 pandemic—A retrospective cohort study from South India
Source: PLoS One. 2025 Jan 24;20(1):e0312993. doi: 10.1371/journal.pone.0312993 (PMC11761102; doi:10.1371/journal.pone.0312993)
Supplement: S1 Table — Footnote: Numbers were too small to be individually analysed, but provided to have a bird’s eye view of the population. COPD–Chronic Obstructive Pulmonary Disease, HIV–Human Immunodeficiency Virus. (DOCX) [file pone.0312993.s002.docx]

| **Details of other comorbidities** | **total number** | **Survivors** | **Non-survivors** |
| --- | --- | --- | --- |
| Hypothyroidism | 13 | 8 | 5 |
| Obesity | 9 | 2 | 7 |
| Bronchial Asthma | 8 | 6 | 2 |
| Tuberculosis | 7 | 1 | 6 |
| COPD | 7 | 3 | 4 |
| Malignancy | 4 | 0 | 4 |
| Stroke | 3 | 0 | 3 |
| Mucormycosis | 2 | 0 | 2 |
| parkinsonism | 2 | 0 | 2 |
| Psychiatric illness | 2 | 1 | 1 |
| Autoimmune disease | 1 | 0 | 1 |
| Seizures | 1 | 0 | 3 |
| HIV | 1 | 1 | 0 |
| Cirrhosis | 1 | 0 | 1 |

**S1 Table. Distribution of other comorbidities between survivors and non-survivors in detail (mentioned as “others” in Table 1)**

**Footnote: Numbers were too small to be individually analysed, but provided to have a bird’s eye view of the population.**

**Legends: COPD – Chronic Obstructive Pulmonary Disease, HIV – Human Immunodeficiency Virus.**
