# Supplementary material for: Prediction of mortality and prioritisation to tertiary care using the ‘OUR-ARCad’ risk score gleaned from the second wave of COVID-19 pandemic—A retrospective cohort study from South India
Source: PLoS One. 2025 Jan 24;20(1):e0312993. doi: 10.1371/journal.pone.0312993 (PMC11761102; doi:10.1371/journal.pone.0312993)
Supplement: S2 Table — The higher percentage of patients using the drugs and injections simply implies that they had more severe disease mandating these interventions. This should not be interpreted that the drugs had actually caused the complications or death. N–total number of participants, %—percentage, O2 –oxygen, <—lesser than. a–Includes both low molecular weight heparin (LMWH) and unfractionated heparin (UFH). (DOCX) [file pone.0312993.s003.docx]

**S2 Table. Treatment details including specific antivirals, steroids and anticoagulants administered, oxygen supplementation and complications that arose in the cohort**

| **Variable** | | **Survivors**  **N (%)**  **325** | **Non-Survivors**  **N (%)**  **292** | **p-value** |
| --- | --- | --- | --- | --- |
| Specific Antivirals | | 89(27.4) | 141(48.3) | <0.001 |
| Dexamethasone | | 178(54.8) | 162(54.5) | 0.859 |
| Methyl prednisolone | | 51(15.7) | 125(42.8) | <0.001 |
| Heparin^a^ | | 215(61.6%) | 269(92.1%) | <0.001 |
| Drug - Ivermectin | | 247(76) | 152(52.1) | <0.001 |
| Drug - Hydroxychloroquine | | 1(0.3) | 2(0.7) | 0.501 |
| Acute Lung Injury /  Acute Respiratory Distress Syndrome | | 0 | 260(89) | <0.001 |
| Acute Kidney Injury | | 1(0.3) | 20(6.8) | <0.001 |
| Sepsis / Multi-Organ Dysfunction | | 0 | 61(20.9) | <0.001 |
| Cardiogenic shock | | 0 | 10(3.4) | <0.001 |
| Mode of O_2_ Delivery | Nil | 179(55.08) | 31(10.32) | <0.001 |
|  | Simple/Non-Rebreather Mask | 138(42.46) | 100(34.36) |  |
|  | High Flow Nasal Cannula | 8(2.46) | 97(33.33) |  |
|  | Mechanical Ventilation | 0(0) | 64(21.99) |  |

The higher percentage of patients using the drugs and injections simply implies that they had more severe disease mandating these interventions. This should not be interpreted that the drugs had actually caused the complications or death.

Legends: N – total number of participants, % - percentage, O_2_ – oxygen, < - lesser than

^a^ – Includes both low molecular weight heparin (LMWH) and unfractionated heparin (UFH)
