# Supplementary material for: Prediction of mortality and prioritisation to tertiary care using the ‘OUR-ARCad’ risk score gleaned from the second wave of COVID-19 pandemic—A retrospective cohort study from South India
Source: PLoS One. 2025 Jan 24;20(1):e0312993. doi: 10.1371/journal.pone.0312993 (PMC11761102; doi:10.1371/journal.pone.0312993)
Supplement: S3 Table — The ‘OUR-ARCad’ risk score of 50, calculated from the sum of the component scores if positive (Table 2) has a sensitivity of 90% and a specificity of 75%. This looks ideal when the referral to medical care is contemplated from a peripheral heath centre. However, when it is in border zone of 38 or more, but less than 50, re-scoring after 3–5 days or estimation of biomarkers if facilities exist is recommended. OUR-ARCad—O–peripheral oxygen saturation in percentage, U–urea in milligram per decilitre, R–Neutrophil lymphocyte ratio, A–age in years, R–Pulse rate in beats per minute, D–Diabetes mellitus, Cad–coronary artery disease / cardiovascular disease, COVID-19 –Corona virus disease 2019. (DOCX) [file pone.0312993.s004.docx]

**S3 Table. Receiver operator curve values for the ‘OUR ARCad’ risk score with their corresponding sensitivity and specificity from second wave of COVID-19 in South India**

| Risk score value | Sensitivity | 1-Specificity |
| --- | --- | --- |
| -1.0000 | 1.000 | 1.000 |
| 4.0000 | 1.000 | 0.826 |
| 11.5000 | 0.996 | 0.702 |
| 15.5000 | 0.996 | 0.694 |
| 19.5000 | 0.996 | 0.683 |
| 27.0000 | 0.991 | 0.589 |
| 34.5000 | 0.978 | 0.408 |
| 38.5000 | 0.978 | 0.377 |
| 42.5000 | 0.974 | 0.340 |
| **50.0000** | **0.896** | **0.245** |
| 57.5000 | 0.722 | 0.147 |
| 61.5000 | 0.661 | 0.128 |
| 65.5000 | 0.557 | 0.094 |
| 72.5000 | 0.243 | 0.023 |
| 76.5000 | 0.235 | 0.023 |
| 80.5000 | 0.070 | 0.000 |
| 88.0000 | 0.022 | 0.000 |
| 93.0000 | 0.000 | 0.000 |

The ‘OUR-ARCad’ risk score of 50, calculated from the sum of the component scores if positive (Table 2) has a sensitivity of 90% and a specificity of 75%. This looks ideal when the referral to medical care is contemplated from a peripheral heath centre. However, when it is in border zone of 38 or more, but less than 50, re-scoring after 3-5 days or estimation of biomarkers if facilities exist is recommended.

Legends: OUR-ARCad - O – peripheral oxygen saturation in percentage, U – urea in milligram per decilitre, R – Neutrophil lymphocyte ratio, A – age in years, R – Pulse rate in beats per minute, D – Diabetes mellitus, Cad – coronary artery disease / cardiovascular disease, COVID-19 – Corona virus disease 2019, - - minus.
